# Retrospondyloptosis of the Spine Secondary to Nonaccidental Trauma

**DOI:** 10.1155/2018/4526560

**Published:** 2018-06-19

**Authors:** T. S. Duffin, S. W. Thomas

**Affiliations:** Department of Pediatrics, Wake Forest School of Medicine, Winston-Salem, NC, USA

## Abstract

Spinal fracture rates from NAT have been reported in <1–3% of spinal injury cases. We present a 13-month-old female who presented with signs of spinal cord injury and was found to have complete retrospondylolisthesis of T12 vertebra and multiple rib fractures in various stages of healing due to NAT. This case reports an extremely severe spinal injury due to NAT of which there are few in the literature and highlights the importance of suspicion of NAT when pediatric patients present with neurologic symptoms and spinal trauma without plausible mechanism of injury.

## 1. Introduction

According to the latest data from the United States in 2015, about 4 million reports of possible child maltreatment were made in that year and about 683,000 children were found to be victims of child maltreatment. While the most common form of child maltreatment is neglect (75%), the second most common is physical abuse, or nonaccidental trauma (NAT), accounting for 17% of all cases [1]. Spinal trauma secondary to NAT is less common than other NAT injuries, and spinal fracture rates from NAT have only been reported in <1–3% of spinal injury cases [2–4]. However, studies looking at the incidence of spinal injury have found that in children less than two years of age, as high as 20–38% of spinal injuries were secondary to NAT accounting for an appreciable number of patients [5, 6]. Spinal cord injury less frequently occurs unless the injury involves listhesis [2, 3]. We present a case of complete spinal dislocation at the thoracolumbar junction resulting in paraplegia secondary to NAT. This case not only highlights the importance of recognizing spinal injuries as a result of NAT, especially in the younger and more vulnerable population.

## 2. Case Description

**Figure 1:**
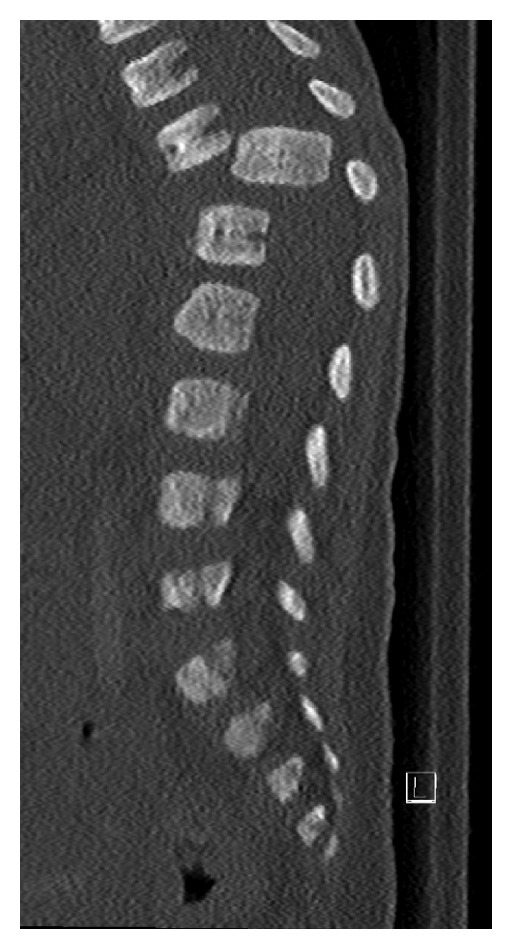
CT of the spine demonstrating complete dislocation of T11 and T12 vertebral bodies. The T12 vertebral body appears to be rotated counterclockwise on axial image and displaced posteriorly into the bony spinal canal, resulting in severe spinal canal stenosis.

**Figure 2:**
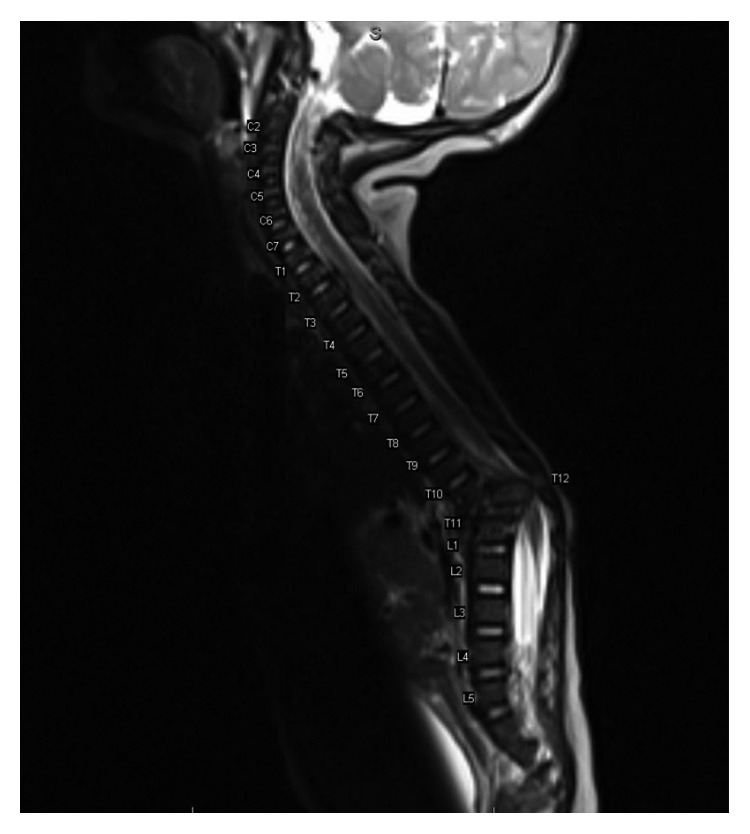
MRI of the spine with separation/avulsion of the T12 vertebral body from the T12 posterior elements. The T12 vertebral body appears to be rotated counterclockwise on axial image and displaced posteriorly into the bony spinal canal resulting in marked compression of the spinal cord along with signal abnormality in the spinal cord just inferior and superior to the compressed T12 segment.

A 13-month-old female presented to the emergency department for urinary retention. She was born at 35 weeks gestation in Mexico with an unknown postnatal hospital course. She was known to have mild gross motor developmental delay diagnosed several months before; caregivers stated that she pulled to stand but did not cruise or walk and babbled but did not have any words. She had no additional past medical history or previous surgeries and no known previous trauma. Upon presentation, the adoptive parents with whom she has lived since very early in her life provided the history. Adoptive parents reported about 2 weeks of fussiness and decreased ability to bear weight on her lower extremities. Parents denied any known trauma at that time. Her weight was <3rd percentile with a Z score of −3.36. She was thin appearing but without signs of dehydration or malnourishment. Examination was notable for 0/5 strength, hypotonia, and areflexia in the bilateral lower extremities as well as hypotonia of the trunk. In addition, she was found to have significant head lag and very severe thoracic kyphosis or gibbus deformity. The patient was first evaluated by outpatient urology after referral from the primary care office for urinary retention. A renal ultrasound was performed and was notable for bilateral moderate hydronephrosis and bladder distension. She was sent to the emergency department for further workup. Laboratory studies in the emergency department yielded normal creatinine for age at 0.3 mg/dl, and urine culture obtained by in and out bladder catheterization grew >100,000 CFU/mL of *Enterococcus faecalis*. Complete blood count and basic metabolic panel were unremarkable, and thyroid stimulating hormone was within normal range. The patient's creatinine kinase was >1200 U/L, six times the upper limit of normal. Spinal CT demonstrated complete retrospondylolisthesis of T12 vertebra (Figure 1). MRI was also performed and showed marked compression of the spinal cord resulting in inability to visualize the cord at the level to T12 with surrounding cord edema (Figure 2). Brain MRI was without abnormality. This imaging also revealed multiple rib fractures in different stages of healing. Dedicated osseous survey showed numerous right and left posterior rib fractures. Liver function tests, lipase, amylase, phosphorous, parathyroid hormone, and vitamin D were obtained to rule out congenital or acquired reasons for predisposition for bony fractures, all of which were found to be within normal range. The patient's findings were consistent with traumatic spinal spondyloptosis with additional findings concerning for physical child abuse. The initial history provided to the medical team included a confusing timeline and described a progressive decline in motor function. That along with a physical examination notable for head lag made this diagnosis initially challenging. Alternative diagnoses of systemic neurodegenerative disorders such as spinal muscular atrophy or other diseases of motor function such as botulism were considered. However, after the imaging, the diagnosis became clear, and concerns for NAT were raised. After confirmation of her diagnosis with imaging, the hospital's child protection team and the state of North Carolina's child protective services were notified. The patient underwent posterior spinal fusion from T11 to L1 to stabilize her spine. She also received intensive rehabilitation after surgery. Unfortunately, she did not regain lower extremity function and remains paraplegic. Her adoptive mother later admitted to shaking the child aggressively by holding her at the level of mid abdomen/lower thoracic back (the area of injury) and throwing her on the floor several weeks prior to presentation.

## 3. Discussion

Spondylolisthesis is defined as the anterior translocation of one vertebra on its adjacent caudal segment. Severity is graded using the Meyerding classification from grade 1–5: grade 1 is 0–25% dislocation, grade 2 is 25–50%, grade 3 is 50–75%, grade 4 is 75–100%, and grade 5, also known as spondyloptosis, refers to complete displacement [7]. Posterior translocation of a vertebra is referred to as posterior spondylolisthesis or retrolisthesis. Retrolisthesis injuries are exceptionally rare and very few case reports of these injuries are reported in the pediatric literature or literature at large [8–10]. Sieradzki and Sarwark report a case that is similar to ours and is compared to only eleven other reports of spinal listhesis due to NAT [11]. Tran and colleagues report an additional T12 fracture dislocation injury due to NAT [12]. As previously noted, spinal trauma makes up an appreciable portion of NAT injuries; however, listhesis is only present is a small portion of those patients. One explanation as to how these types of injuries occur in children is the difference between adult and pediatric anatomy. The vertebra of children are unique in that they have growth centers of the neurocentral synchondrosis and both superior and inferior aspects of the vertebral body which can result in growth plate fractures and injuries from trauma, similar to growth plates of long bones. The fractures then allow for a free moving vertebral body, which can more easily displace. The theorized mechanism of injury is best described by Carrion et al. who note the combination of axial load, flexion, and rotation needed to produce the type of injury in our patient [13]. In particular, traumatic spondyloptosis results from high energy forces with the majority of the literature citing injuries from car crashes, falls from great heights, crush injuries and so on. [14]. Thus, in the absence of such high energy mechanisms, listhesis is highly suggestive of NAT, and therefore physicians should have a high index of suspicion for NAT in these cases. As noted previously, spinal cord injury is a rare comorbidity in patients with spinal injuries due to NAT. However, our patient's presentation highlights the features of acute spinal compression: symmetric paralysis of limbs, urinary retention or incontinence, and a “sensory level” or circumferential boundary below which there is loss of sensation. Hyperreflexia and positive Babinski sign, traditionally thought of as signs of spinal cord pathology, may not be present in cases of acute and severe cord compression. Instead, limbs may be flaccid and areflexic as was the case in our patient. In patients with the above physical examination findings and concern for spinal cord compression, CT is the initial preferred imaging and should be obtained urgently [15].
